# Omics-based exploration of biomarkers and therapeutic targets in olfactory neuroblastoma

**DOI:** 10.1007/s12672-026-05299-0

**Published:** 2026-05-28

**Authors:** Ning Deng, Zehao Chen, Carolina Oi Lam Ung, Yunfeng Lai, Menghuan Song, Hao Hu

**Affiliations:** 1 State Key Laboratory of Mechanism and Quality of Chinese Medicine, University of Macau, Macao, China; 2 Centre for Pharmaceutical Regulatory Sciences, Institute of Chinese Medical Sciences, University of Macau, Macao, China; 3 Department of Public Health and Medicinal Administration, Faculty of Health Sciences, University of Macau, Macao, China; 4 School of Public Health and Management, Guangzhou University of Chinese Medicine, Guangzhou, Guangdong Province China

**Keywords:** Olfactory neuroblastoma, Omics, Biomarker, Therapeutic target, Target therapy

## Abstract

**Introduction:**

Olfactory Neuroblastoma (ONB) is a rare and aggressive malignant tumor with a complex, heterogeneous pathogenesis. Although various omics studies have been conducted for ONB recently, there is still no omics evidence summary for ONB. Therefore, this study systematically reviews and synthesizes evidence across multiple omics layers for identifying biomarkers and therapeutic targets of ONB, aiming to advance the translation of molecular insights into clinical applications, including subtype classification, targeted therapy, and drug development.

**Methods:**

We conducted a systematic review following the PRISMA 2020 guidelines and registered with PROSPERO (ID: CRD420251128889). Five databases were searched up to 31st May 2025 for original studies that applied single- or multi-omics analyses to ONB. Risk of bias was assessed using the JBI Case Series Checklist.

**Results:**

A total of 24 studies were included, covering genomics (11/24), transcriptomics (5/24), proteomics (1/24), and multi-omics integration (7/24). Genomic studies identified recurrent mutations (e.g., TP53, IDH2), chromosomal alterations (e.g., 1p, 3p, 13q), and pathway-level changes (e.g., PI3K/AKT/mTOR, chromatin remodeling). Transcriptomic studies revealed Basal, Neural, and Mesenchymal subtypes, with NEUROD1 and EZH2 serving as key biomarkers. For proteomics, one immunohistochemistry-focused study reports markers such as Trk proteins and GRP78. Multi-omics studies integrated subtyping, tumor-origin hypotheses, and immune landscape characterization, supporting biomarkers such as IDH2, NEUROD1, and EZH2, and highlighting heterogeneity. However, there is very limited functional validation (only one study) and limited AI/ML usage (two studies), despite their potential to assist with small cohorts and multimodal integration.

**Conclusion:**

This review consolidates current evidence on candidate biomarkers and therapeutic targets for ONB, highlighting the vital role of omics-based approaches in elucidating its molecular mechanisms. To advance the field, future research should focus on standardizing methods, validating findings in larger, more diverse groups, and integrating multi-omics techniques with AI–driven analyses to enhance diagnostic precision and develop targeted treatments.

**Supplementary Information:**

The online version contains supplementary material available at 10.1007/s12672-026-05299-0.

## Introduction

Olfactory Neuroblastoma (ONB) is a rare and aggressive malignant tumor originating from the olfactory neuroepithelium [1, 2]. It primarily affects the nasal cavity and olfactory nerve region; therefore, as the tumor progresses, patients may experience headache, facial numbness, and other neurological symptoms [3]. In addition to nasal endoscopy, the diagnosis of ONB involves advanced imaging techniques, such as MRI and CT scans, to assess tumor size and location [4]. Also, a biopsy is essential for confirming the diagnosis and is generally performed following imaging [5]. The pathogenesis of ONB is characterized by complexity and heterogeneity. Even though staging systems such as Kadish and Hyams are frequently used for ONB staging, it’s hard to make accurate prognostic predictions based solely on such staging systems [6–9]. Treatment of ONB typically involves multidisciplinary approaches, including surgical resection, radiotherapy, and chemotherapy [4, 5]. However, it may be difficult to achieve complete resection due to the tumor’s location, the extent of local invasion, or distant nodal/metastatic disease [4]. Previous studies have demonstrated that combining chemotherapy with surgery and/or radiotherapy yields better overall survival than a single treatment modality [10]. It remains challenging to achieve a complete cure, since the prognosis depends on multiple complex clinical factors [11].

Since understanding molecular profiling and mechanisms plays a significant role in improving strategies for diagnosis, classification, and treatment, bioinformatics has become a crucial tool in oncology, integrating biology, computer science, and statistics to analyze large biological datasets [12]. Bioinformatics can leverage diversified omics approaches, including genomics, transcriptomics, proteomics, immunomics, and multi-omics, etc., to reveal molecular mechanisms and identify biomarkers and therapeutic targets for oncology as ONB [13–16]. As omics-based explorations of ONB can vary across omics layers, there is a need to synthesize omics evidence to consolidate the understanding of ONB.

Previously, Kaur et al. conducted a systematic review mainly focusing on genomic and epigenetic studies of ONB, identifying genetic mutations and alterations associated with the tumor, which revealed mutations in key genes such as TP53 and CDKN2C [17]. However, their analyses lack the integration of multi-omics data such as transcriptomics and proteomics. More recently, Demir et al. presented a valuable, comprehensive overview that integrates ONB research with multi-omics analyses to identify key molecular pathways, immune evasion mechanisms, and two distinct molecular subtypes in ONB, offering new perspectives for precision diagnosis and therapy [18]. But as a narrative review, it lacks a standardized systematic screening process and quality assessment.

Moreover, neither of the previous works evaluated the clinical translational potential of the reported findings, nor did they investigate the emerging role of artificial intelligence (AI) and machine learning (ML) methods in ONB research. Therefore, to bridge this significant research gap, this study presents a comprehensive systematic review and synthesizes evidence across multiple omics layers to identify potential biomarkers and therapeutic targets of ONB. Unlike previous reviews, we implement quality assessment and specifically highlight the experimental validation supporting these molecular targets. And we also synthesize the application of AI/ML methodologies. We aim at providing a highly reliable framework that advances the translation of molecular insights into clinical applications, including subtype classification, targeted therapy, and drug development.

## Methods

This systematic review was conducted to investigate current developments in identifying biomarkers and therapeutic targets of ONB using omics approaches, following the PRISMA 2020 guidelines, and was registered with PROSPERO (ID: CRD420251128889) [19].

### Study search

A comprehensive search was conducted in 5 databases (PubMed, Embase, Scopus, Web of Science, and Cochrane) from the inception to 31st May 2025. The search strategy combined disease-specific terms (“olfactory neuroblastoma” / “esthesioneuroblastoma”) with a comprehensive set of omics-related keywords covering genomics, transcriptomics, proteomics, epigenomics, and metabolomics. Controlled vocabulary terms (MeSH/Emtree), free-text terms, Boolean operators, and database-specific field tags were applied consistently across databases. The search was restricted to articles published in English and Chinese. The complete search strings, query dates, and results are provided in Supplementary Material 1 to ensure transparency and reproducibility.

### Study selection

All retrieved citations were imported into EndNote, and duplicates were removed. Pre-determined eligibility criteria were applied. We only included those studies focused exclusively on ONB or from which ONB-related outcomes could be extracted separately. Studies that involved other cancer types simultaneously were excluded. Studies employing any type of omics analysis (single or multi-omics) were eligible for inclusion. We considered all model types, including human subjects, animal models, and cell lines. Only original research articles were considered. Reviews, meta-analyses, editorials, grey literature, preprint servers, and conference abstracts were excluded. All texts were screened manually by two independent investigators (ND and ZC). Discrepancies were resolved through discussions between all authors.

### Data extraction

Relevant data were systematically retrieved from each included study using a standardized form that captured sample information, omics data, data analysis methods, study findings, and functional validation. Data extraction was performed by one investigator (ND), and subsequently cross-checked for accuracy and completeness by a second independent investigator (ZC). All discrepancies were resolved through discussion.

### Risk of bias assessment

JBI Critical Appraisal Checklist for Case Series was applied to assess the risk of bias [20]. Two independent reviewers (ND and ZC) conducted the assessment for each included study. Each of the ten items on the checklist was evaluated separately, and responses were categorized as “Yes,” “No,” or “Unclear” according to JBI guidance. Discrepancies between reviewers were resolved through discussion and consensus. In cases where consensus could not be reached, a third reviewer (HH) was consulted to adjudicate.

## Results

### Selected studies

**Fig. 1:**
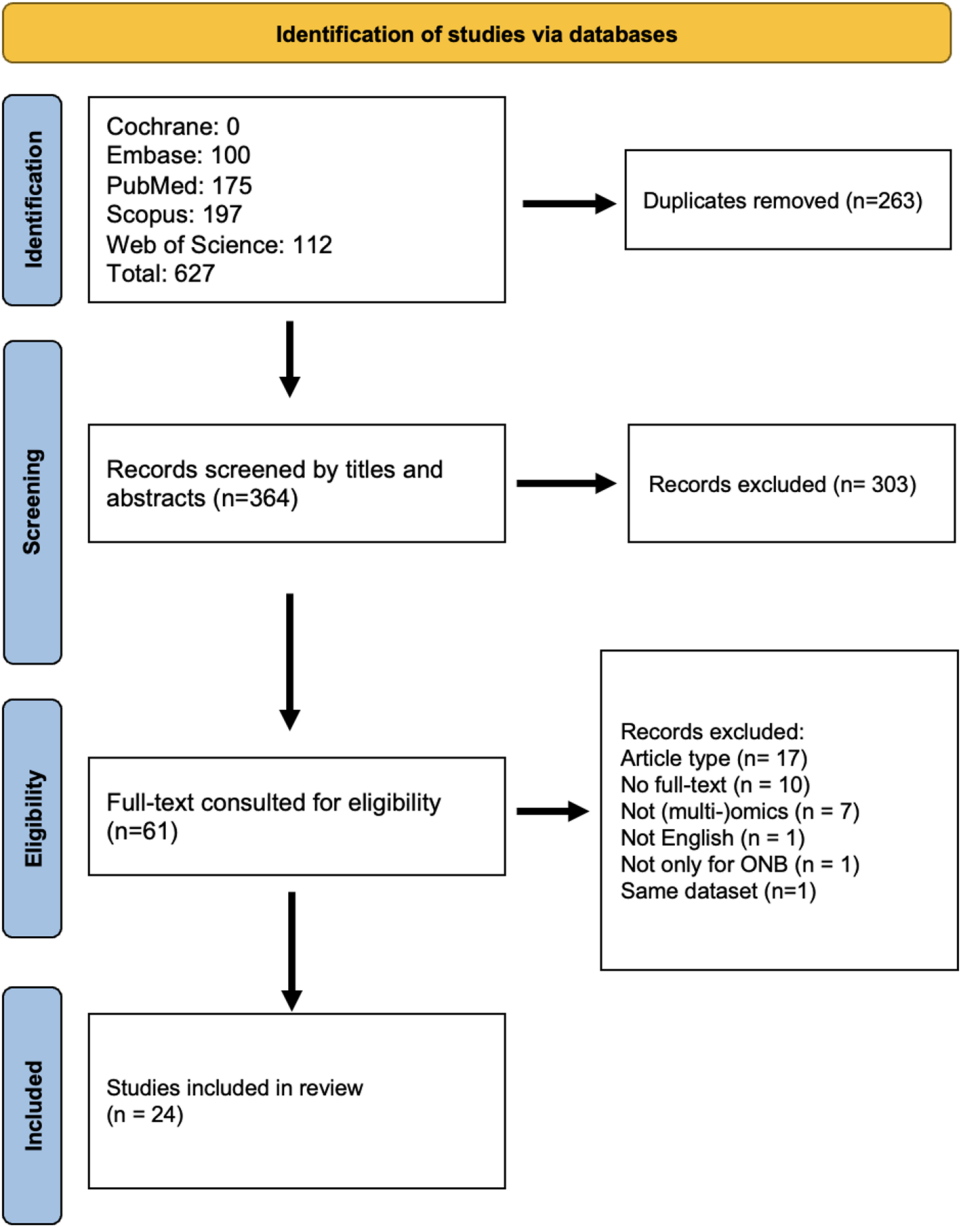
PRISMA flow chart

Totally, we identified 627 studies from the original search. After removing 263 duplicates, 364 studies were filtered by title and abstract; 303 were removed. 61 articles were reviewed by full text. After applying the inclusion and exclusion criteria, 24 studies were finally included in our systematic review (Fig. 1).

### Risk of bias assessment

**Fig. 2:**
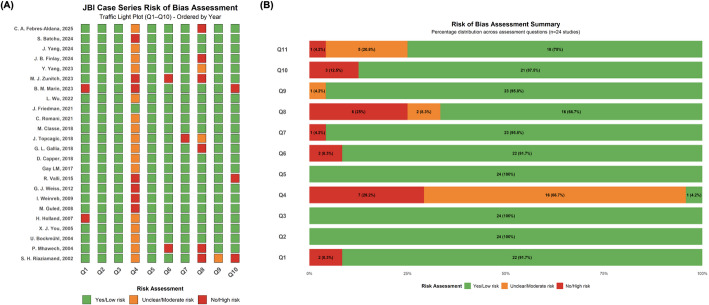
Risk of bias assessment using JBI Critical Appraisal Checklist for Case Series. (Q1: Were there clear criteria for inclusion in the case series? Q2: Was the condition measured in a standard, reliable way for all participants included in the case series? Q3: Were valid methods used for identification of the condition for all participants included in the case series? Q4: Did the case series have consecutive inclusion of participants? Q5: Did the case series have complete inclusion of participants? Q6: Was there clear reporting of the demographics of the participants in the study? Q7: Was there clear reporting of clinical information of the participants? Q8: Were the outcomes or follow up results of cases clearly reported? Q9: Was there clear reporting of the presenting site(s)/clinic(s) demographic information? Q10: Was statistical analysis appropriate? Q11: The overall risk of bias assessment? )

We applied the JBI Critical Appraisal Checklist for Case Series to evaluate the methodological quality of the included studies (Fig. 2). Among the 24 studies, the majority (18/24) had low risk of bias, indicating generally reliable reporting and methodological rigor. Five studies were identified as moderate risk, primarily due to incomplete reporting of clinical outcomes or lack of statistical analysis. One study was assessed as high risk of bias due to being a single-case report with limited methodological detail and the absence of statistical analysis. Given the rarity of ONB, no studies were excluded based on these assessments. However, findings derived primarily from moderate- or high-risk studies were interpreted with strict caution. In conclusion, most studies demonstrated acceptable quality, but variability in reporting and analysis indicated the need for more standardized and robust study designs in ONB research.

Importantly, evidence synthesis in this review was interpreted in light of study-level risk-of-bias assessments. Key recurrent findings, such as IDH2 mutations, ONB molecular subtypes, and NEUROD1/EZH2 expression patterns, were primarily supported by studies assessed as low risk of bias, increasing confidence in their reproducibility. In contrast, findings derived mainly from small case reports or studies with a moderate to high risk of bias were interpreted with caution and not used to draw strong conclusions regarding clinical translation.

### Characteristics of included studies

Of all the included studies, the first publication was released in 2002 [21]. 14/24 studies were published before 2020 [21–34]. Table 1 shows the basic characteristics of the included studies. It is worth noting that Bockmühl et al. and You et al. reported CGH analyses of the same patient cohort from the same institution; therefore, these two publications were merged and treated as a single study (*n* = 12 patients) in our data synthesis. All included studies reported sample size, source, and type, with most samples derived from formalin-fixed paraffin-embedded (FFPE) or fresh/frozen tumor tissues. Regarding demographic information, the majority of included studies provided sex ratios and ages (both 18/24), indicating that ONB patients are typically middle-aged, with reported mean or median ages ranging from 29 to 62 years. Males accounted for 50% to 65% of participants, with some studies reporting higher male ratios or exclusively male cases. Most studies described therapeutic interventions, primarily involving surgical resection, often combined with radiotherapy or chemotherapy. Also, most studies provided staging information using Kadish or Hyams systems (21/24).

**Table 1:** Basic characteristics of included studies

Author, year	Sample size	Country	Sample type	Sex (male ratio)	Age (mean)	Stage	Therapy	Study type	Experimental validation
S. H. Riaziamand, 2002	3	Germany	FFPE ENB tumor tissue	33.3%	57	Kadish C	All resection, some of those received RT and Chemotherapy	Small case series	No
U. Bockmühl, 2004 / X. J. You, 2005	22 (12 patients)	Germany	FFPE	58.3%	49	Morita B-D	Surgery + adjuvant stereotactic radiotherapy	Small case series	No
P. Mhawech, 2004	24 (5 ONB)	Switzerland	Fresh-frozen tumor tissue	NM	NM	Hyams grade I-IV	NM	Small cohort	No
H. Holland, 2007	1	Germany	Fresh surgical tumor tissue	100%	43	Kadish stage C; UCLA T4; Hyams grade III-IV	Surgical resection + postoperative radiotherapy	Case report	No
M. Guled, 2008	13	USA	FFPE	61.5%	45.5	Kadish stage 2–3	NM	Small case series	No
I. Weinreb, 2009	20	Canada	FFPE	65%	49.3	Hyams grades I-IV	Surgery, radiation, chemoradiation (mixed; details not fully specified)	Large cohort molecular study	No
G. J. Weiss, 2012	1	USA	FFPE	100%	29	Kadish C (inferred)*	Surgery, radiation, chemotherapy	Case report	No
R. Valli, 2015	11 (10 patients)	Italy	FFPE and fresh surgical tumor tissues	50%	54.2	Kadish B-C; Hyams I-III	NM	Small case series	No
L M. Gay, 2017	41	USA	FFPE	NM	50.9	Kadish 3–4 / D	NM	Large case series	No
D. Capper, 2018	66	Germany, Switzerland, UK, USA	FFPE	50%	62 (Median)	Hyams I-IV	NM	Large cohort molecular study	No
M. Classe, 2018	59	France	Fresh-frozen and FFPE tumor tissues	NM	47.7	Kadish A-D; Hyams I-IV	Surgery ± radiation ± chemotherapy	Large cohort molecular study	No
G. L. Gallia, 2018	14	USA	Fresh-frozen tumor tissues and matched blood	78.6%	NM	NM	Surgery ± radiation ± chemotherapy	Small cohort	No
J. Topcagic, 2018	23	USA	FFPE tumor tissues	43.5%	57.7	NM	Surgery ± radiation ± chemotherapy ± target therapy	Large case series	No
J. Friedman, 2021	14	USA	FFPE tumor tissues	50%	50	Kadish A-D; Hyams II-IV	All received surgery, some received rt/rct, 1 received preoperative rct	Small cohort	No
C. Romani, 2021	32	Italy	FFPE tumor tissues	53.1%	53.5 (median)	TNM I-IV; Hyams I-IV	All received surgery, 84.4% received postoperative rt/rct	Large cohort molecular study	No
L. Wu, 2022	82	China	FFPE tumor tissues	73.2%	46 (median)	Kadish A-C; Hyams I-IV	Surgery ± rt/ct	Large cohort molecular study	No
B. M. Marin, 2023	1	USA	Fresh tumor tissues (primary and spinal metastasis from one patient)	100%	50	Hyams IV	Surgery + rt + ct	Case report	No
Y. Yang, 2023	19	China	FFPE tumor tissues	57.9%	NM	Kadish A-C; Hyams I-IV	Surgery with no preoperative treatment	Small cohort	No
M. J. Zunitch, 2023	16	USA	Fresh-frozen and cryopreserved tumor tissues	NM	NM	Hyams I-IV	Surgery with no preoperative treatment	Small cohort	No
S. Batchu, 2024	18	USA	GEO dataset (GSE118995); Fresh-frozen tumor tissues	55.6%	47.5	Hyams I-IV	Surgery with no preoperative treatment	Small cohort	No
J. B. Finlay, 2024	Mouse model (*n* = 26); human tumor (*n* = 14)	USA	FFPE and fresh-frozen human tumor tissues; mouse tumors and GBC-derived organoids	NM	NM	Hyams I-IV	NA	Study type: Preclinical model study / Small cohort	Yes (in vivo)
J. Yang, 2024	10 (scRNA-seq) / 23 (bulk RNA-seq) / 30 (IHC validation)	China	Fresh and FFPE tumor tissues; normal olfactory mucosa	NM	NM	Kadish A-D; Hyams I-IV	NM	Large cohort molecular study	No
C. A. Febres-Aldana, 2025	27 ONB of total 76 patients	USA	FFPE tumor tissues	59%	57 (median)	NM	NM	Large cohort molecular study	No

*:For studies that did not specifically report tumor staging using Kadish, Morita, or Hyams systems, staging was inferred based on clinical descriptions such as tumor extent, recurrence, and presence of metastases

These cases are marked as ‘inferred’ in Table 1

### Genomics

Of all the included studies, genomics was the most frequently applied omics approach, adopted in 11 included studies (Table 2) [21, 22, 24–26, 28–30, 33, 35, 36]. The earliest genomics study on ONB was published in 2002 [21]. Early studies mainly used comparative genomic hybridization (CGH) and fluorescence in situ hybridization (FISH) to detect chromosomal alterations. And more advanced approaches, such as array-CGH, single nucleotide polymorphism (SNP) arrays, whole-exome sequencing (WES), whole-genome sequencing (WGS), and targeted next-generation sequencing (NGS) were adopted in the decade, which enhanced the resolution detection of structural rearrangement, copy number variations (CNVs), and point mutations, helping us gain a more comprehensive understanding of ONB’s genomic landscape.

Across the included studies, multiple recurrent chromosomal alterations and gene mutations were identified. Frequently mentioned chromosomal gains include the following regions: 8q, 13q, 17q, 20q, and 22q, especially in high-stage tumor. Chromosomal losses at 1p, 3p, 4q, 6q, 10q, and 13q were also commonly observed, with 1p21-p31 deletion repeatedly associated with poor prognosis. At the gene level, TP53 mutations have been reported in multiple studies. While IDH2 mutations, specifically R172, were detected in 18.3% ONB cases, which is associated with high-stage tumor and poor prognosis. Alterations in the PI3K/AKT/mTOR pathway (e.g., PIK3CA, PTEN, NF1, IGF1R, IRS1) and chromatin remodeling genes (e.g., SMARCA4, DNMT3B) were also mentioned repeatedly. G. L. Gallia et al. demonstrated DMD gene loss in 86% of cases in their study, indicating it as a potential tumor suppressor gene [33]. Currently, the overall genomic landscape of ONB remains heterogeneous, but overlapping findings across studies highlight multiple recurrent alterations with potential diagnostic and therapeutic value.

**Table 2:** Studies applying genomics approaches to identify potential biomarkers or therapeutic targets of ONB

Author, year	Methods	Results
S. H. Riaziamand, 2002 [21]	CGH	For all 3 samples: *Gain*: 8q24.1-qter, 15q25-qter, 22q-qter, Chr 19 *Loss*: 4q-qter For some of the samples: *Gain*: 1p32-pter, 9q34.1-qter, 10q24.3-qter, 6p-pter *Loss*: 13q-qter
U. Bockmühl, 2004 [22] / X. J. You, 2005 [24]	CGH	Chromosomal loss: Entire chromosome: 3, 4, 5, 10 Chromosome arm: 6q, 13q, 18q, 21q High-frequency regions: 1p21–p31, 3p12–p14, 3p21–p23, 3q13, 3p/q, 4p/q, 4p14–p15, 5p14, 5p/q, 5q, 6q14–q23, 8p21, 8q21, 8q23, 9p21–p23, 9p, 9q22–q33, 10p/q, 11p14, 12p11.2–p12, 12q21, 13q, 14q21, 18q, 21q21 Pronounced loss: 3p12–p13, 3p22, 3p26, 3q13, 3q24–q25, 4p13–p15, 4q, 10q26, 13q21–q23 Chromosomal gains: Entire chromosome: 17, 19, 22 Chromosome arm: 1q, 16p, 17q, 20q High-frequency regions: 1q12, 1q24–q32, 2q22–q32, 7q11.2, 8q, 9q34, 11q12–13, 11q13, 14q, 16p11, 16q, 17q12, 17q21, 17q24–q25, 17p13, 19p/q, 20p, 20q12–q13.1, 22q11.2, 22q13 Pronounced gains: 1p33–p36, 1p34, 1q23–q31, 7p21, 7q31, 9p23–p24, 17q11–q22, 17q24–q25, 19, 20, 20p, 20q13, 22q13 Metastatic chromosomal alterations: Loss: 5p/q, 6q, 7q, 11p/q, 15q Gain: 1p32–p34, 1q12, 2p22–p24 Poor prognosis-related chromosomal alterations: Loss: 1p21–p31, 4p/q, 5p/q, 6q, 7q31–q32, 9p, 11p/q, 15q21 Gain: 1q12, 8q, 20q
H. Holland, 2007 [25]	GTG banding, M-FISH, locus-specific FISH, SNP	Applying GTG banding, M-FISH, and FISH: *High-frequency chromosomal loss*: 2q37 (first-reported), 6q22, 6q24, 21q22 (first-reported), 22q13 *Other chromosomal loss*: 1p12–p33, 3p11–p25, 3q25–q26, 6p21, 10q26, 11q23, 15q26, 20q11.1–q12 Applying SNP: *Chromosomal loss*: 2q37.3, 3q27.2, 10q26.11, 21q22.1 *Chromosomal gain*: 2q14.3, 13q33.3, 13q34, 17q12, 17q21.31, 17q25.3, 22q13.1, 22q13.31, 22q13.33 Of those related to known oncogenes: BRCA1 (17q21.31), ERBB2 (17q12), FLI1 (11q24.3), PDGFB (22q13.1), Cyclin D1 (11q13)
M. Guled, 2008 [26]	aCGH	Chromosomal gains: *High-frequency chromosomal gains (≥ 50%)*: 13q14.2–q14.3, 13q31.1, 20q11.21–q11.23, 5q35, 13q, 20q *Other chromosomal gains (≥ 20%)*: 1p36.31, 1p35.3, 4p12–p15.31, 4q12, 4q21–q35.2, 7q11.22–q21.11(including LIMK1 and FZD9 gene), 9p13.3, 10p12.31, 12q23.1–q24.31, 13q34 (including TFDP1 and CUL4A gene), 22q12.1, Xp/q Chromosomal loss: *High-frequency chromosomal loss (≥ 50%)*: Xp21.1, 2q31.1, 2q33.3, 6q16–q22 *Other chromosomal loss (≥ 20%)*: 2q37.1, 4p13, 5q31.2, 6p21.33, 6p12.3, 15q11.2–q24.1, 18q12.2–q12.3, 19q12–q13, 22q11.1–q12.1 Kadish stage 3 tumor: 28.5 chromosomal alterations per case The entire chromosome gains or loss is more frequent (e.g. 20,21, 22, X) High-frequency alterations: *Gain*: 13q14.2–q14.3, 13q31.1, 20q11.21–q11.23 *Loss*: Xp21.1, 2q31.1, 2q33.3, 6q16–q22 Potentially related genes: *Oncogenes*: LIMK1(7q11.2), FZD9(7q11.2), BRK(20q13.3), TFDP1 and CUL4A(13q34) *Tumor suppressor genes*: ADAM23(2q33.3), FOXO3 and CCNC(6q21–q22)
G. J. Weiss, 2012 [28]	WGS	Early-stage related mutations: TP53, MAP4K2, TAOK2 Late-stage or metastasis related mutations: KDR, MYC, SIN3B, NLRC4
R. Valli, 2015 [29]	aCGH	No consistent or high-frequency chromosomal mutation regions were found. Chromosomal gains: Chr19, 20q13 Chromosomal loss: Part of Chr15 and Chr22
LM. Gay, 2017 [30]	CGP, NGS	High-frequency gene mutations: TP53 (17% cases, Chr17) PIK3CA, NF1, PTEN, PIK3R2, TSC1, RICTOR: *related to PI3K/mTOR pathway* CDKN2A, CDKN2B, CDKN2C, CDK6: *related to cell cycle* CTNNB1, ARID1A, KDM5C, SMARCA4: *related to chromatin remodeling* IDH2, ATM, MLH1, TET2: *related to DNA repairing and metabolism*
G. L. Gallia, 2018 [33]	WES, WGS, SNP array analysis, FISH	Gene loss: DMD (Dystrophin): 86%, probably tumor suppressor gene LAMA2: 7% Gene mutation: TTN: 14%, missense mutation
L. Wu, 2022 [35]	IHC, RT-PCR	IDH2 mutations were found in 15 cases (18.3%). Mutations sites concentrated at R172 (exon 4) *IDH2 mutation is significantly related to*: High Kadish stage, High Hyams level, High Ki-67 index, low expression of neuroendocrine markers (e.g. chromogranin A, synaptophysin, CD56, S100) *Kaplan-Meier analysis showed*: The overall survival (OS) of patients with IDH2 mutations is significantly lower than that of patients with wild-type IDH2 High Hyams level and High Ki-67 index is related to poor prognosis. *Multivariate Cox regression analysis showed*: IDH2 mutation and Hyams level are independent factors for poor prognosis.
B. M. Marin, 2023 [36]	Targeted Exome Sequencing	PI3K/AKT/mTOR pathway related genes: IGF1R (15q26.3): mutations in both primary lesion and metastatic lesion IRS1 (2q36.3): mutations only in metastatic lesion with a high frequency (95.8%) Chromatin remodeling related genes: SMARCA4 (19p13.2): mutations in both primary lesion and metastatic lesion (p.D1314N) DNA repairing related genes: DNMT3B (20q11.21): mutations only in metastatic lesion MLH3 (14q24.3): mutations only in primary lesion Other key genes: PLCG2 (16q23.3): mutations only in metastatic lesion GRM3 (7q21.11): mutations only in metastatic lesion FGFR1 (8p11.23): mutations only in primary lesion AR (androgen receptor)(Xq12): mutations in both primary lesion and metastatic lesion

### Transcriptomics

Among the included studies, 5 studies employed transcriptomics approaches (Table 3) [23, 37–40]. The earliest transcriptomics study by Mhawech et al. used RT-PCR and TaqMan technology to assess hASH1 mRNA expression [23]. Even though this work focused on a single gene, it represents a foundational example of candidate gene transcriptomics and presented the diagnostic potential of transcriptional markers in ONB. Following a long gap in transcriptomics publications on ONB, multiple studies in recent years have conducted comprehensive transcriptomic profiling using bulk RNA-seq, single-cell RNA-seq, and spatial transcriptomics, integrating these approaches with advanced bioinformatics tools such as GSEA, ssGSEA, CIBERSORTx, and scVI.

Across the included studies, the molecular subtyping of ONB was consistently reported, dividing it into 3 subtypes: Basal, Neural, and Mesenchymal. The Basal subtype was associated with high Ki-67 index, low S100 expression, poor differentiation and prognosis. The Neural subtype showed mature neuroendocrine features and better outcomes. The Mesenchymal subtype proposed by Yang et al. was correlated to immunosuppression and angiogenesis, showing the potential effectiveness of anti-angiogenic therapies [40]. Among the studies, NEUROD1 is consistently identified as a biomarker of ONB, distinguishing it from other sinonasal tumors, such as SNUC. Also, its expression supports the hypothesis that ONB originates from neurogenic globose basal cells (npGBCs) in the olfactory epithelium.

**Table 3:** Studies applying transcriptomics approaches to identify potential biomarkers or therapeutic targets of ONB

Author, year	Methods	Results
P. Mhawech, 2004 [23]	RT-PCR, TaqMan	hASH1 gene localization: 12q22-q23 hASH1 mRNA expression: Positive in 4/5 ENB cases, of which 3 were well-differentiated ENB, 1 was poorly differentiated ENB. Negative in all 19 PDT cases. The expression level of hASH1 mRNA is negatively correlated with the degree of ENB differentiation: the worse the differentiation, the higher the expression.
C. Romani, 2021 [37]	Gene Expression Profiling, Microarray Data Processing, Differential Expression Analysis, GSEA, ssGSEA	CK (Pan-cytokeratin) positive and CK negative: 1777 differentially expressed genes were identified, of which 1015 were upregulated in CK positive tumor and 762 were downregulated in CK positive tumor. *BUB1* is one of the most significantly upregulated genes. The pathways significantly enriched in metastatic patients include: TGF-β binding and signaling pathways, epithelial-stromal transition (EMT), IFN-α reaction, angiogenesis, the IL2-STAT5 and IL6-JAK-STAT3 signaling pathways These pathways are significantly associated with a shorter disease-free survival (DFS). Subtype classification: According to CK expression and Ki-67 index, ONB could be classified into: *Basal type*: Positive CK, high Ki-67 expression, low S100 expression, with poor prognosis. *Neural type*: Negative CK, low Ki-67, high S100 expression, and well-differentiated.
S. Batchu, 2024 [38]	Bulk RNA sequencing, CIBERSORTx deconvolution	Basal subtype tumor: the CD4 activated memory T cells increased significantly Neural subtype tumor: the resting dendritic cells increased The mutation rate of IDH2 is higher in basal type tumors (37.5% vs. 0%, *P* = 0.04) Although there were differences in the composition of immune cells, there was no significant difference in survival rate (*P* = 0.5).
J. B. Finlay, 2024 [39]	Bulk RNA-seq, Single-cell RNA-seq, Spatial transcriptomics, spatial transcriptomics	ONB is highly similar to small cell lung cancer (SCLC) in terms of transcriptional characteristics, lineage trajectories and immune microenvironments. *NEUROD1 and POU2F3 are mutually exclusive expressions.* High-frequency chromosomal alterations: TP53, RB1, MYC, MYCL, chr17p, chr8q, chr1p34(MYCL sites) GBC (globose basal cells) have been verified as *potential origin cells* of ONB. The immune microenvironment presented that *ONB might be insensitive to immunotherapy*. DLL3, SEZ6, BCL2, SSTR2, and UCHL1 are upregulated in Neural subtype ONB, which have potential therapeutic value.
J. Yang, 2024 [40]	scRNA-seq, Bulk RNA-seq	ONB could be classified into 3 molecular subtypes: *Neural*,* Basal*,* and Mesenchymal*. Neural: the CNV levels are relatively high, originates from olfactory neural progenitors Basal: the CNV levels are relatively low, originates from GBC, sensitive to cell cycle inhibitor, with a worst prognosis in three subtypes Mesenchymal: the CNV levels are relatively low, originates from HBCs and sustentacular cells, highly related to immunosuppression and angiogenesis, sensitive to anti-angiogenic drugs, with a best prognosis in three subtypes

### Proteomics

Proteomic research in ONB remains limited, with only one study systematically investigating the protein expression patterns of ONB [27]. Using an immunohistochemistry approach on 20 ONB cases, I. Weinreb et al. found high expression of Trk-A (90%), Trk-B (85%), and GRP78 (90%), while p75NRT showed focal staining in 60% of cases [27]. Trk-A and GRP78 showed a statistically significant positive correlation with overall and recurrence-free survival; however, the small sample size may limit the conclusions. There was no marker correlated with Hyams grade according to this study. The consistent expression of Trk proteins suggests it as a possible oncogene in ONB.

### Multi-omics integration

In recent years, multi-omics studies in ONB have emerged, showing a trend towards more comprehensive molecular profiling. Among the 24 included studies, 7 studies employed multi-omics strategies, integrating data from at least two omics layers such as genomics, epigenomics, transcriptomics, proteomics, or immunomics, using techniques such as WES, RNA-seq, DNA methylation profiling, CNV analysis, and IHC (Table 4) [31, 32, 34, 41–44]. These multi-omics studies enabled molecular subtyping of ONB (e.g., Basal, Neural, Mesenchymal), the identification of subtype-specific biomarkers (e.g., IDH2, EZH2, NEUROD1), and the characterization of the tumor microenvironment, providing insights into tumor origin and heterogeneity. Classe et al. and Yang et al. identified Basal subtype tumors as poorly differentiated, IDH2-mutated, and immune-infiltrated, while Neural subtype tumors showed mature neuroendocrine features and a better prognosis [32, 42]. Yang et al. also presented the potential of target therapy for ONB, as their study exhibited multiple high-frequency mutated genes, such as CTNNB1 and ZNRF3 [42]. Studies by Zunitch et al. further supported the neural origin hypothesis, identifying GBCs as likely precursors and highlighting markers like NEUROD1 and EZH2 [43]. The multi-omics approaches have significantly advanced ONB studies by revealing overlapping molecular features across studies and suggesting clinically potential targets.

**Table 4:** Studies applying multi-omics approaches to identify potential biomarkers or therapeutic targets of ONB

Author, year	Omics types	Methods	Results
D. Capper, 2018 [31]	Genomics, Epigenomics	Genome-wide DNA methylation profiling, CNV analysis, NGS, IHC, Sanger sequencing	DNA methylation analysis reclassified 66 cases of ONB into four categories, among which only 64% were true “Core ONB”. Core ONB has typical histological structure, low Hyams level, high-frequency whole chromosome loss (Chr 1–4, 8–10, 12) and TP53/DNMT3A mutations. IDH2-mutated tumors present a CIMP phenotype. It is recommended to assist in diagnosis through methylation profiling and IDH2 detection.
M. Classe, 2018 [32]	Genomics, Transcriptomics, Epigenomics, Proteomics	WES, RNA-Seq, DNA methylation profiling, PCA, GO enrichment analysis, KEGG, IHC	Multi-omics analysis divided ENB into *neural subtype and basal subtype*. About one-third of the basal subtype ENB carrying *IDH2 R172 mutation* and exhibiting a *CpG island methylation phenotype* (E-CIMP). The basal subtype has poor differentiation, high proliferation, strong immune infiltration, and poor prognosis. The neural subtype shows more mature neurological features and better prognosis.
J. Topcagic, 2018 [34]	Genomics, Transcriptomics, Proteomics	NGS, Sanger sequencing, Whole-genome RNA microarray, IHC, FISH, CISH	Upregulated genes: CD24, SCG2, IGFBP-2 Totally 146 genes were upregulated in ONB Downregulated genes: ABCA8, GHR Totally 183 genes were downregulated in ONB Mutated genes: TP53, CTNNB1, EGFR, APC, cKIT, cMET, PDGFRA, CDH1, FH, SMAD4
J. Friedman, 2021 [41]	Genomics, Transcriptomics, Immunomics	Targeted DNA sequencing. Whole transcriptome RNA sequencing, TMB analysis, Immune cell deconvolution from RNA-seq	Gene loss: ERBB2, CCND1, CDK6, PTCH1, PMS2, MLH1, MSH2 Gene deletion: NOTCH1 Gene frameshift: TET2 Gene missense: KRAS Q61R Low TMB Low PD-1, PD-L1, and CTLA-4 expression 190 mutations were detected. Clinically relevant mutations are mainly concentrated in the following pathways: RTK, Cell cycle, P53/apoptosis, GLI, MMR
Y. Yang, 2023 [42]	Genomics, Immunomics	WES, CNV detection, Ki-67 immunohistochemistry	A total of 929 non-synonymous mutations were detected, and 18/19 cases (94.74%) had mutations. High-frequency gene mutation: CTNNB1 (16%) ZNRF3 (16%) EGFR, JARID2, KMT2B, KMT2C, NSD1, PIK3CA, SH2B3, UBR5 (each 11%) TP53 (5%) Copy number variation (CNV): 10/19 cases (52.63%) had CNV, mainly copy-number loss. COL11A1 and PRAMEF17 are absent (each 21%) High-frequency abnormal pathways: WNT pathway, RAS pathway Tumor mutational burden (TMB): No significant differences between the low-grade group and high-grade group (Hyams) Immunomics: PD-L1 expression: Only 1/15 cases showed an expression of > 1% in the tumor area CD68 macrophages: The low-grade group was significantly higher than the higher-level group Ki-67 index: The high-grade group was significantly higher
M. J. Zunitch, 2023 [43]	Transcriptomics, Immunomics	Bulk RNA sequencing, Single-cell RNA sequencing, GSEA, IHC, IF	Cell Origin and Differentiation Trajectory: ONB closely resembles npGBCs in the olfactory epithelium. ONB cells express NEUROD1, which distinguishes them from SNUC (NEUROD1-negative). ONB contains a highly proliferative EZH2 + cell population, suggesting EZH2 as a potential therapeutic target. ONB cells exhibit divergent differentiation: One branch toward neuronal maturation (from npGBC to iOSN), while the other branch toward sustentacular-like differentiation (HES1-driven) Immunogenomic Markers: NEUROD1, EZH2, HES1, KRT8/18/17 Molecular Mechanisms and Therapeutic Potential: ONB might originate from npGBCs and retains developmental regulatory pathways. EZH2 is a promising therapeutic target (has been validated in other cancers). HES1-driven bifurcation may explain ONB heterogeneity.
C. A. Febres-Aldana, 2025 [44]	Transcriptomics, Immunohistochemistry	IHC, PCA, GSEA, TMA, RNA-seq data mining	ONB-related key results: NEUROD1-dominant subtype (100% of ONB cases) POU2F3-negative (0% expression) ASCL1 low or negative YAP1 low or negative High neuroendocrine differentiation (NE-high) (96% of ONB cases, mean H-score ≈ 266) *Strong expression of conventional neuroendocrine markers (cNEM)*: Synaptophysin, Chromogranin A, CD56, INSM1 Distinct from other tumor types (e.g., SNUC, OC) in nNEM expression profile *RNA-seq validation*: ONB classified as “neural subtype ENB” with high NEUROD1 and low POU2F3/YAP1 expression NEUROD1 positively correlates with neuroendocrine differentiation, which YAP1 negatively correlates with. NEUROD1 is a useful diagnostic marker to distinguish ONB from SNUC and OC

### Functional validation

While multi-omics studies had expanded our understanding of ONB, experimental validation of these computational findings remained limited. Notably, only one of the included studies conducted experimental functional validation by engineering mouse models and performing organoid transplantation to track lineage trajectories and investigate the molecular heterogeneity of ONB (Table 5), thereby providing direct biological evidence for ONB’s lineage plasticity and cellular origin [39].

**Table 5:** Summary of wet lab experiments in Finlay et al

Workflow	Experimental modules	Materials	Objectives	Key findings and conclusions
Disease modelling	Genetically Engineered Mouse Models (GEMMs)	Rb1/Trp53/Myc (RPM) mice; RPM-Ascl1 knockout (RPMA) mice; intranasal Ad-Cgrp-Cre adenovirus	To develop models of ONB to interrogate genetic alterations, cell of origin, lineage-related transcription factors, and cell fate plasticity.	RPM mice develop high-grade metastatic ONB exhibiting a NEUROD1 + immature neuronal phenotype. ASCL1 loss in RPM ONB leads to emergence of non-neuronal histopathology, including a POU2F3 + microvillar-like state.
Cell of origin validation	In vitro organoid culture and allografts	Methimazole (MMZ)-treated mouse olfactory epithelium; purified KIT+ globose basal cells (GBCs); SCID/beige immunodeficient mice	To specifically address if GBCs can transform into ONB.	Ex vivo Cre-transformed GBC-derived organoids transplanted into mice developed palpable tumors that histologically resembled primary RPM ONB. This demonstrates that GBCs can serve as a permissive ONB cell of origin.
Tumor evolution track	Lineage tracing and cellular barcoding	Cre reporter “Ai9” (Rosa26-LSL-tdTomato) mice; lentiviral CellTag library	To track the ability of GBC-derived ONB allografts to become neuronal or non-neuronal tumor populations and determine whether individual cells undergo phenotypic switching.	The vast majority of tdTomato+ fate-mapped cells are GBCs or their derivatives. CellTagged clones in the allografts span multiple transcriptionally distinct states, confirming ONB exhibits plasticity between neuronal and non-neuronal states.
Clinical translation & targeting	Single-Cell and spatial transcriptomics	10X Genomics scRNA-seq (RPM/RPMA tumors and allografts, human ONB); NanoString GeoMx spatial transcriptomics (12 human ONB specimens)	To better understand transcriptional heterogeneity in mouse ONB and its relationship to human tumors, profile immune populations, and examine SCLC therapeutic targets.	Mouse and human ONBs exhibit transcriptional similarity to SCLC. Human ONB harbours intratumoral cell fate heterogeneity, an immune-cold tumor microenvironment, and shared expression of therapeutic targets like DLL3, SEZ6, and BCL2.

### AI/ML usage

Two studies applied machine learning-based computational tools to analyse ONB transcriptomics data, including CIBERSORTx, a support vector regression tool used for immune cell deconvolution, and scVI, a probabilistic deep learning model applied for unsupervised clustering [38, 39]. These AI-driven approaches greatly facilitated the characterization of the microenvironment and the subtype classification of ONB.

## Discussion

Our systematic review shows that, between 2002 and 2025, the omics landscape in ONB research demonstrated a clear evolutionary trajectory of methodological advancement: from early genomic descriptions of chromosomal alterations to the recent integration of single-cell, spatial, and multi-omics data. Cross-omics integration is essential for implementing precision oncology. In our included studies, recurrent IDH2 mutations and distinct methylation profiles were identified in isolated single-omics studies and were connected by multi-omics integration, demonstrating that IDH2 mutations lead to a specific CpG island promoter methylation phenotype (CIMP) [31, 32, 35]. And this is linked to the “Basal” subtype, a high-risk, poorly differentiated transcriptomics subtype of ONB [31, 32]. Similarly, the PI3K/AKT/mTOR signaling pathway was identified as a vulnerability across multiple independent studies [30, 36]. Furthermore, high-resolution single-cell and spatial transcriptomics have converged with bulk RNA-seq data to identify transcription factors such as NEUROD1 and epigenetic regulators such as EZH2 not only as biomarkers of ONB but also as critical drivers of lineage plasticity and tumor origin [39, 40, 43]. This cross-omics convergence helps transform our understanding of ONB into a disease with clinically actionable molecular characteristics, providing valuable insights into the clinical translation of diagnostic algorithms, precise molecular subtyping, and targeted therapies.

Since ONB shares significant histopathological and immunophenotypic overlap with other sinonasal small round cell tumors, especially sinonasal undifferentiated carcinoma (SNUC), it’s necessary to integrate omics-derived biomarkers into routine diagnosis and pathology. Zunitch et al. identified EZH2 as a biomarker of ONB cell proliferation, particularly in high-grade ONB [43]. The expression of EZH2, along with NEUROD1, related ONB to globose basal cells (GBCs) of the olfactory epithelium, providing insight into tumor origin and helping differential diagnosis. A study by Febres-Aldana et al. demonstrated that ONB consistently expresses high levels of NEUROD1 and lacks expression of POU2F3, ASCL1, and YAP1, which could distinguish ONB from SNUC and other sinonasal small round cell tumors, supporting the utility of these transcription factors as diagnostic tools [44]. Given the consistent and mutually exclusive expression patterns across ONB and other tumors, such transcriptional regulators and genetic mutations showed strong potential for clinical translation. Future studies are encouraged to validate the sensitivity and specificity of these biomarkers in larger cohorts. And we strongly recommend implementing an IHC panel that incorporates NEUROD1 alongside conventional neuroendocrine markers as the first step in a molecular-pathological diagnostic process.

Beyond initial diagnosis, molecular subtyping of ONB has been conducted in multiple studies. Although Hyams grading system is widely used in ONB classification, it lacks molecular support [9]. The multi-omics identification of “Basal”, “Neural”, and “Mesenchymal” subtypes represents a shift for ONB risk stratification [32, 40]. However, it’s impractical to perform whole-transcriptome sequencing for every patient. To achieve true clinical utility, we propose that these complex multi-omics subtypes be integrated into the clinical diagnostic process using highly specific biomarkers instead. In addition to its potential as a diagnostic biomarker discussed above, IDH2 also contributes to the molecular subtyping of ONB. Specifically, tumors harboring IDH2 R172 mutations and exhibiting elevated EZH2 expression or a high Ki-67 proliferation index can be clinically classified as the “Basal” subtype, indicating a poorly differentiated status and early metastasis risk, alerting to the need for more aggressive therapies and surveillance [31, 32, 35, 43]. Conversely, IDH2-wildtype tumors with mature neuroendocrine features, such as high NEUROD1 expression, represent the more “Neural” subtype [32]. And the “Mesenchymal” subtype was characterized by immunosuppression and angiogenesis, which introduces a potential rationale for incorporating anti-angiogenic evaluations into the diagnostic process [40]. By adapting a classification framework that combines IDH2 status, an IHC panel (NEUROD1, EZH2, Ki-67), and Hyams grade, clinicians can effectively perform precision prognostic stratification of ONB.

Therapeutic strategies of ONB were also expected to shift towards precision oncology. Gay et al. and Marin et al. identified multiple high-frequency gene mutations in the PI3K/AKT/mTOR pathways, including PIK3CA, IGF1R, and IRS1, consistently suggesting the potential of the PI3K/AKT/mTOR pathway as a clinically actionable avenue [30, 36]. Early evidence reported an illustrative case of a response to combined targeted therapies (sorafenib following everolimus, an inhibitor targeting the mTOR pathway) [45]. Additionally, targeted therapies appeared promising, particularly in the Neural subtype. Further supporting this, another case report described a successful disease control using ^177^Lutetium-DOTA-octreotate (^177^Lu-DOTA-TATE), a radiolabeled somatostatin analogue [46]. Octreoscan was used in this case to detect upregulated SSTR2 expression, which is considered a specificity of the neural subtype [39, 47]. However, the effectiveness of immunotherapy may not be as expected. The characterizations of ONB as low-TMB expression and lack of upregulation of immunotherapy target genes suggest limited utility. Nonetheless, Batchu et al. and Yang et al. compared different subtypes of ONB and indicated that the Basal subtype may respond better to immune checkpoint inhibitors than other subtypes, as activated CD4 memory T cells were significantly increased in this subtype [35, 38, 40]. Despite the absence of standardized treatment guidelines, accumulating molecular studies and case reports have shown promise in therapeutic strategies.

Among the included studies, only one study conducted systematic experimental validation, underscoring a critical gap between omics-based discovery and mechanistic or translational confirmation in the current ONB landscape. While numerous candidate drivers and therapeutic targets have been proposed, it is important to recognize that not all omics-identified alterations are equally ready for functional investigation. In particular, targets such as EZH2 and key components of the PI3K/AKT/mTOR signaling pathway are supported by convergent evidence across genomics, transcriptomics, and multi-omics studies, and have also been functionally implicated in related neuroendocrine malignancies. These features position them as higher-priority candidates for experimental validation, compared with low-frequency or subtype-restricted alterations that lack reproducibility across cohorts. To move beyond associative findings, future studies should adopt robust in vitro and in vivo platforms, including patient-derived organoids (PDOs) and genetically engineered mouse models (GEMMs), such as those established by Finlay et al. [39] Using these systems, targeted perturbation strategies (e.g., CRISPR/Cas9-mediated knockout or pharmacologic inhibition) can be applied to directly assess tumor dependency, lineage plasticity, and therapeutic vulnerability. Such prioritised functional pipelines will be essential for advancing ONB research from molecular characterization toward mechanism-driven clinical translation.

Maximizing the utility of multi-omics data in rare cancers such as ONB will inevitably require advanced computational support; however, current applications of AI and machine learning remain largely descriptive rather than predictive [38, 39]. This limitation is primarily driven by extremely small cohort sizes, data heterogeneity, and the absence of standardized multi-center datasets, which collectively constrain the feasibility of complex end-to-end deep learning models. Under these practical constraints, near-term AI/ML efforts are likely to be most impactful when applied to well-defined, task-specific problems, such as transcriptomic subtype classification, immune cell deconvolution from bulk RNA-seq data, and integrative pattern recognition across small multi-omics cohorts. In contrast, fully automated clinical decision-making or large-scale outcome prediction models should currently be regarded as exploratory rather than immediately attainable goals [48]. As data-sharing initiatives expand and harmonized ONB cohorts become available, more sophisticated multimodal frameworks integrating histopathology, genomics, and clinical variables may become feasible. Until then, strategically targeted AI/ML applications that align with data scale and biological context represent the most realistic and productive path forward.

### Research limitations

This systematic review is subject to several limitations. First, the predominance of small cohorts and case series underscores the importance of cautious interpretation, particularly when findings originate from studies with limited methodological rigor. Second, although multi-omics investigations are increasingly reported, most included studies employed single-omics strategies, thereby limiting integrative analyses and cross-validation across molecular layers. Third, most studies were constrained by limited sample sizes and infrequent functional validation, which may compromise the robustness and external validity of the findings, reflecting a persistent disconnect between molecular discovery and mechanistic elucidation. Fourth, pronounced heterogeneity in analytical frameworks, significance thresholds, and data preprocessing pipelines was evident, impeding direct comparability and quantitative synthesis. Finally, while certain studies incorporated advanced computational methodologies, the adoption of AI and ML techniques remains nascent, underscoring a critical opportunity for methodological advancement in future research.

## Conclusion

This systematic review synthesizes current evidence on potential biomarkers and therapeutic targets in olfactory neuroblastoma (ONB). By integrating findings from genomics, transcriptomics, proteomics, and epigenetic landscape, current evidence converges on key drivers, such as IDH2 mutations, NEUROD1/EZH2 expression, and the PI3K/AKT/mTOR signaling pathway, which could define the origin of ONB and the molecular subtypes (Basal, Neural, Mesenchymal). Taken together, the principal bottleneck in translating ONB omics discoveries is not the lack of candidate targets, but rather the absence of prioritized functional validation pipelines and appropriately scaled computational strategies tailored to rare cancers. Crucially, to precisely bridge the gap between bench discovery and clinical application, we suggest translating complex omics signatures into an actionable diagnostic panel to guide precision oncology. Therefore, to fully realize the translational potential, future research should prioritize functional validation in vivo or in vitro of current targets. Furthermore, strategically leveraging advanced AI-driven tools will be essential for integrating multi-modal data, overcoming the limitations of small cohorts, and accelerating the development of personalized therapeutic frameworks for ONB.

## Supplementary Information

Below is the link to the electronic supplementary material.

Supplementary Material 1.

## Data Availability

No datasets were generated or analysed during the current study.
