# Supplementary material for: Omics-based exploration of biomarkers and therapeutic targets in olfactory neuroblastoma
Source: Discov Oncol. 2026 May 28;17:1090. doi: 10.1007/s12672-026-05299-0 (PMC13407801; doi:10.1007/s12672-026-05299-0)
Supplement: Supplementary file 1 — Supplementary Material 1. [file 12672_2026_5299_MOESM1_ESM.docx]

## **Supplementary Material 1. Search Strategy**

For each database, controlled vocabulary terms (such as MeSH for PubMed and Emtree for Embase) were systematically combined with free-text terms using appropriate Boolean operators (AND/OR) and field tags. Database-specific syntax was retained without post hoc modification to reflect native indexing structures.

| Database | Query date | Search strategy | Query results | language |
| --- | --- | --- | --- | --- |
| PubMed | 31^st^ May 2025 | (((("esthesioneuroblastoma, olfactory"[MeSH Terms]) OR (olfactory neuroblastoma)) OR (olfactory neuroblastomas)) OR (esthesioneuroblastoma)) AND ((((((((((((((((((("genomics"[MeSH Terms]) OR ("transcriptome"[MeSH Terms])) OR ("proteomics"[MeSH Terms])) OR ("epigenomics"[MeSH Terms])) OR (genomics)) OR (transcriptomics)) OR (proteomics)) OR (genetics)) OR (epigenomic)) OR (pharmacogenomics)) OR (phylogenomic)) OR (epigenetic)) OR (epigenetics)) OR (miRNA)) OR (histone)) OR (methylation)) OR (acetylation)) OR (metabolomics)) OR (metabolomics[MeSH Terms])) | 175 | English, Chinese |
| Embase | 31^st^ May 2025 | ('esthesioneuroblastoma'/exp OR 'esthesioneuroblastoma':ab,ti,kw OR 'olfactory neuroblastoma*':ab,ti,kw) AND ('genomics'/exp OR 'transcriptomics'/exp OR 'proteomics'/exp OR 'metabolomics'/exp OR 'epigenetics'/exp OR 'genomic*':ab,ti,kw OR 'transcriptomic*':ab,ti,kw OR 'proteomic*':ab,ti,kw OR 'genetics*':ab,ti,kw OR 'epigenomic*':ab,ti,kw OR 'pharmacogenomic*':ab,ti,kw OR 'phylogenomic*':ab,ti,kw OR 'epigenetic*':ab,ti,kw OR 'mirna*':ab,ti,kw OR 'microrna'/exp OR 'histone*':ab,ti,kw OR 'methylat*':ab,ti,kw OR 'acetylat*':ab,ti,kw OR 'metabolomic*':ab,ti,kw) | 143 | English, Chinese |
| Web of Science | 31^st^ May 2025 | ((((((((((((((TS=(genomic*)) OR TS=(transcriptomic*)) OR TS=(proteomic*)) OR TS=(metabolomic*)) OR TS=(epigenetic*)) OR TS=(genetic*)) OR TS=(epigenomic*)) OR TS=(pharmacogenomic*)) OR TS=(phylogenomic*)) OR TS=(mirna*)) OR TS=(microrna*)) OR TS=(histone*)) OR TS=(methylat*)) OR TS=(acetylat*)) AND ((TS=(esthesioneuroblastoma*)) OR TS=(olfactory neuroblastoma*)) | 112 | English, Chinese |
| Scopus | 31^st^ May 2025 | (TITLE-ABS-KEY ( genomic* ) OR TITLE-ABS-KEY ( transcriptomic* ) OR TITLE-ABS-KEY ( proteomic* ) OR TITLE-ABS-KEY ( metabolomic* ) OR TITLE-ABS-KEY ( epigenetic* ) OR TITLE-ABS-KEY ( genetic* ) OR TITLE-ABS-KEY ( epigenomic* ) OR TITLE-ABS-KEY ( pharmacogenomic* ) OR TITLE-ABS-KEY ( phylogenomic* ) OR TITLE-ABS-KEY ( mirna* ) OR TITLE-ABS-KEY ( microrna* ) OR TITLE-ABS-KEY ( histone* ) OR TITLE-ABS-KEY ( methylat* ) OR TITLE-ABS-KEY ( acetylat* )) AND (TITLE-ABS-KEY ( esthesioneuroblastoma* ) OR TITLE-ABS-KEY ( olfactory AND neuroblastoma* )) | 197 | English, Chinese |
| Cochrane | 31^st^ May 2025 | (((esthesioneuroblastoma) OR (olfactory neuroblastoma) OR (esthesioneuroblastomas) OR (olfactory neuroblastoma)):ti,ab,kw) AND (((genomics) OR (genomic) OR (transcriptome) OR (proteomics) OR (proteomic) OR (epigenomics) OR (epigenomic) OR (transcriptomics) OR (transcriptomic) OR (pharmacogenomics) OR (pharmacogenomic) OR (phylogenomic) OR (epigenetic) OR (epigenetics) OR (miRNA) OR (miRNAs) OR (microRNA) OR (microRNAs) OR (histone) OR (histones) OR (methylation) OR (acetylation) OR (methyl) OR (methylate) OR (methylated) OR (methylates) OR (methylating) OR (methylations) OR (methylational) OR (methylator) OR (methylators) OR (methyls) OR (acetyl) OR (acetylate) OR (acetylated) OR (acetylates) OR (acetylating) OR (acetylations) OR (acetyls) OR (metabolomics) OR (metabolomic)):ti,ab,kw) | 0 | English, Chinese |
